# *ZP1*-Y262C mutation causes abnormal zona pellucida formation and female infertility in humans

**DOI:** 10.3389/fgene.2024.1407202

**Published:** 2024-06-20

**Authors:** Guangyi Cao, Lina Yu, Junshun Fang, Ruixin Shi, Huijun Li, Feifei Lu, Xiaoyue Shen, Xiangyu Zhu, Shanshan Wang, Na Kong

**Affiliations:** ^1^ Center for Reproductive Medicine and Obstetrics and Gynecology, Nanjing Drum Tower Hospital, Affiliated Hospital of Medical School, Nanjing University, Nanjing, China; ^2^ Center for Molecular Reproductive Medicine, Nanjing University, Nanjing, China; ^3^ Key Laboratory of Reproductive Medicine of Guangdong Province, Guangzhou, China; ^4^ Center for Reproductive Medicine and Obstetrics and Gynecology, Joint Institute of Nanjing Drum Tower Hospital for Life and Health, College of Life Science, Nanjing Normal University, Nanjing, China

**Keywords:** ZP1, zona pellucida, oocyte, reproduction, infertility

## Abstract

Defective oocyte maturation is a common cause of female infertility. The loss of the zona pellucida (ZP) represents a specific condition of impaired oocyte maturation. The extracellular matrix known as the ZP envelops mammalian oocytes and preimplantation embryos, exerting significant influence on oogenesis, fertilization, and embryo implantation. However, the genetic factors leading to the loss of the ZP in oocytes are not well understood. This study focused on patients who underwent oocyte retrieval surgery after ovarian stimulation and were found to have abnormal oocyte maturation without the presence of the ZP. Ultrasonography was performed during the surgical procedure to evaluate follicle development. Peripheral blood samples from the patient were subjected to exome sequencing. Here, a novel, previously unreported heterozygous mutation in the *ZP1* gene was identified. Within the *ZP1* gene, we discovered a novel heterozygous mutation (*ZP1* NM_207341.4:c.785A>G (p.Y262C)), specifically located in the trefoil domain. Bioinformatics comparisons further revealed conservation of the *ZP1*-Y262C mutation across different species. Model predictions of amino acid mutations on protein structure and cell immunofluorescence/western blot experiments collectively confirmed the detrimental effects of the *ZP1*-Y262C mutation on the function and expression of the ZP1 protein. The *ZP1*-Y262C mutation represents the novel mutation in the trefoil domain of the ZP1 protein, which is associated with defective oocyte maturation in humans. Our report enhances comprehension regarding the involvement of ZP-associated genes in female infertility and offers enriched understanding for the genetic diagnosis of this condition.

## Introduction

Infertility in humans is characterized by the inability to achieve conception following 1 year of unprotected sexual intercourse, with comparable contributions from both males and females to its etiology. Common genetic causes of female infertility include polycystic ovary syndrome (PCOS), oocyte maturation defects (OMD), or premature ovarian failure (POF) (Yatsenko and Rajkovic, 2019). Women with oocyte maturation defects have varying degrees of defects in oocyte maturation, including oocyte absence, oocyte immaturity, follicular atresia, or failure of oocytes to be fertilized. The diplotene stage of prophase I, commonly referred to as the germinal vesicle (GV) stage, represents the period of arrest for primary oocytes within the ovary. Following a surge in luteinizing hormone (LH), GV-stage oocytes initiate meiosis, leading to the extrusion of the first polar body(Pb1), and subsequently halt at the metaphase II (MII) stage of the second meiotic division (Eppig et al., 1996).

Zona pellucida (ZP) deficiency is a specific phenomenon associated with oocyte maturation defects. The ZP is a glycoprotein matrix that forms during oocyte growth and remains present around the oocyte and early embryos until embryo hatching (Avella et al., 2014). Within the follicle, the ZP forms a physical barrier separating the oocyte from the surrounding granulosa cells. Concurrently, the ZP presents in the follicular fluid in anticipation of the arrival of sperm (Carino et al., 2001; Hasegawa and Koyama, 2007). The main constituents of the ZP in humans are four proteins (hZP1-hZP4), each featuring zona pellucida domain (ZPD), transmembrane domain (TMD), conserved furin cleavage site (CFCS), and a signal sequence. Notably, the trefoil domain is exclusive to ZP1 and ZP4 (Jovine et al., 2002; Litscher and Wassarman, 2020a). The composition of the ZP differs between mice and humans. In mice, the ZP is composed of three proteins (mZP1-mZP3). Female mice with homozygous mutations in *Zp2* or *Zp3* produce oocytes lacking ZP or degenerate oocytes, resulting in complete infertility (Liu et al., 1996; Rankin et al., 1996; Rankin et al., 2001). Female mice with homozygous *Zp1* mutations have a reduced number of implanted embryos and decreased litter size (Rankin et al., 1999).

On the other hand, in the context of assisted reproductive treatments, the color, thickness, and refractive index of the zona pellucida (ZP) are often used as indicators of oocyte quality. However, the genetic factors underlying ZP defects remain unclear, and recent advancements in high-throughput sequencing, particularly whole-exome sequencing (WES), have made it possible to identify pathogenic gene mutation sites (Dai et al., 2019; Yuan et al., 2019; Cao et al., 2020; Luo et al., 2020; Okutman et al., 2020; Loeuillet et al., 2022; Pujalte et al., 2023).

Here, through whole-exome sequencing, a novel heterozygous mutation (*ZP1* NM_207341.4:c.785A>G (p.Y262C)) situated at the trefoil domain of the ZP1 protein was identified in this investigation. This novel mutation, which we report here, affects the trefoil domain of ZP1. Despite the presence of several large follicles, the patient in this case exhibited oocytes with missing ZP and was unable to undergo fertilization. Confirmation of the functional consequences of this newly identified mutation on the ZP1 protein was obtained through subsequent bioinformatic analysis and protein expression experiments.

## Materials and methods

### Ethical approval

Approval for this study was granted by the Ethics Committee of Nanjing Drum Tower Hospital, Affiliated Hospital of Medical School, Nanjing University (2021-384-01). The embryos analyzed were obtained from the Reproductive Medicine Center of Nanjing Drum Tower Hospital, Nanjing University Medical School. All participants providing clinical samples for this study provided informed consent.

### Searching for genetic mutations

Peripheral blood samples were collected from the patients for the extraction of genomic DNA. Fragmentation and library preparation were performed on the extracted DNA. The resulting DNA sequences were aligned with the human genome (hg19) to evaluate the coverage and quality of the specific regions of interest. Identified variants underwent bioinformatics analysis to determine their pathogenicity. The classification of variants adhered to the guidelines established by the Human Genome Variation Society (HGVS) (website: http://varnomen.hgvs.org/). Criteria for assessing variant pathogenicity were established based on the variant interpretation standards and guidelines developed by the American College of Medical Genetics and Genomics (Richards et al., 2015; Kalia et al., 2017). It should be acknowledged that our approach may have limitations in identifying potentially pathogenic variants within deep intronic or gene regulatory regions with microvariants (deletions or insertions) spanning less than 10 base pairs. Moreover, our method is not suitable for detecting specific genomic structural variations such as complex rearrangements, large-scale deletions or duplications, inversions, dynamic mutations, or translocations.

### Conservation analysis and protein modeling

The IBS 2.0 software was employed to visualize the mutation sites in the *ZP1* gene. Conservation analysis of ZP1 amino acids across multiple species, including mouse, macaque, rat, horse, rabbit, and human, was conducted using the Align feature on the UniProt website (https://www.uniprot.org/). Using the SWISS-MODEL website (https://swissmodel.expasy.org), schematic diagrams of the wild-type (WT) and mutant (*ZP1*, NM_207341.4, c.785A>G (p.Y262C)) ZP1 proteins were generated. The model was based on the reference template (3nk3.1.A.pdb).

### Functional impact prediction of mutant proteins

The impaired function of *ZP1*-Y262C mutations in proteins, including disease-associated variants, was predicted using the PolyPhen-2 tool.PolyPhen-2 (website: http://genetics.bwh.harvard.edu/pph2/), integrates sequence, structure, and conservation information to evaluate the effect of mutations on protein function. It provides predictive scores categorizing the mutations as benign, possibly deleterious, or deleterious. Once loading the amino acid sequences of the *ZP1*-WT and *ZP1*-Y262C mutant into these softs and interpreting the results, insights into the impact of the mutation on protein function can be derived. Higher scores, closer to 1, indicate a greater degree of functional impairment.

### Changes in *ZP1* expression levels

To underscore the significance of *ZP1* in the progression of oocyte maturation and embryonic development, we aimed to elucidate the temporal expression patterns of *ZP1* across diverse species at distinct developmental stages. Leveraging the single-cell transcriptome repository, we reexamined the spatiotemporal dynamics of *ZP1* mRNA expression throughout different embryonic milestones, including 2-PN-zygote, 2-Cell-embryo, 4-Cell-embryo, 8-Cell-embryo, blastocyst, early inner cell mass, and late inner cell mass stages (E-MTAB-7078) (Boroviak et al., 2018). Additionally, the mRNA translatome data was used to reanalyzed dynamic expression of *Zp1* mRNA associated with ribosomes in mouse oocytes and embryos (GSE165782) (Xiong et al., 2022). In summary, RNA sequencing library preparation and sequencing steps were performed using the Smart-seq2 protocol (Picelli et al., 2014) for samples at different time points. Initially, M2 medium was used to perform two washes on oocytes or embryos. Subsequently, cell lysis was conducted using Rnase inhibitor-containing lysis buffer. Following this, cDNA libraries were prepared and subjected to high-throughput sequencing. The alterations in ribosome-associated RNA molecules were depicted using RPF (ribosome-associated RNA expression) profile plots obtained through the utilization of ultra-low-input Ribo-seq (Ribo-lite) methodology (Xiong et al., 2022).

### Plasmid construction, cell culture, and transfection

Human *ZP1* and its mutant form (p.Thy262Cys) were generated and integrated into the eukaryotic expression vector pcDNA3.1, with His and GFP tags attached to the C-terminus and N-terminus of ZP1. These constructs were synthesized by Genescript (Nanjing, China). HEK293T cells were maintained in DMEM (Gibco, 12,491,015) supplemented with 100 mg/mL streptomycin/penicillin (Beyotime Biotechnology, C0222) and 10% fetal bovine serum (FBS, Clark, FB25015) under 37°C and 5% CO2. Upon reaching 80% confluence, cells were transiently transfected with Lipofectamine 2000 reagent (Invitrogen, 11,668,019). Following transfection, cells were rinsed with PBS and incubated in serum-free medium for 2 days prior to harvesting.

### Protein immunoblotting

Preparation of cell lysates and supernatants involved the utilization of RIPA cell lysis buffer (Thermo Scientific, 89,900). Protein concentrations were determined using the BCA Protein Assay Kit (Thermo Scientific, 23,227). Subsequently, proteins underwent separation on a 10% SDS-PAGE gel and subsequent transfer onto a PVDF membrane. To mitigate non-specific binding, a 2-h room temperature incubation in 5% non-fat milk diluted in Tris-buffered saline containing 0.05% Tween-20 was performed to block the membrane. The PVDF membrane was subsequently incubated overnight at 4°C with diluted antibodies for GAPDH (Abclonal, AC002) and ZP1 (Santa Cruz, sc-365435). After three washes with TBST, at room temperature, the membrane was incubated with a horseradish peroxidase (HRP)-conjugated secondary antibody (diluted 1:10,000) for 1 h in TBST. The proteins were visualized using chemiluminescence (Bio-Rad) after another three rounds of washing the membrane with TBST.

### Statistical analysis

Between-group mean comparisons were conducted using one-way analysis of variance (ANOVA). All experiments were carried out three times, and the results are expressed as SEM (mean ± standard error). Statistical significance was determined at a *p*-value less than 0.05. SPSS 16.0 was utilized for all statistical analyses.

## Results

### Phenotype of a patient with zona pellucida-deficient oocytes

**FIGURE 1:**
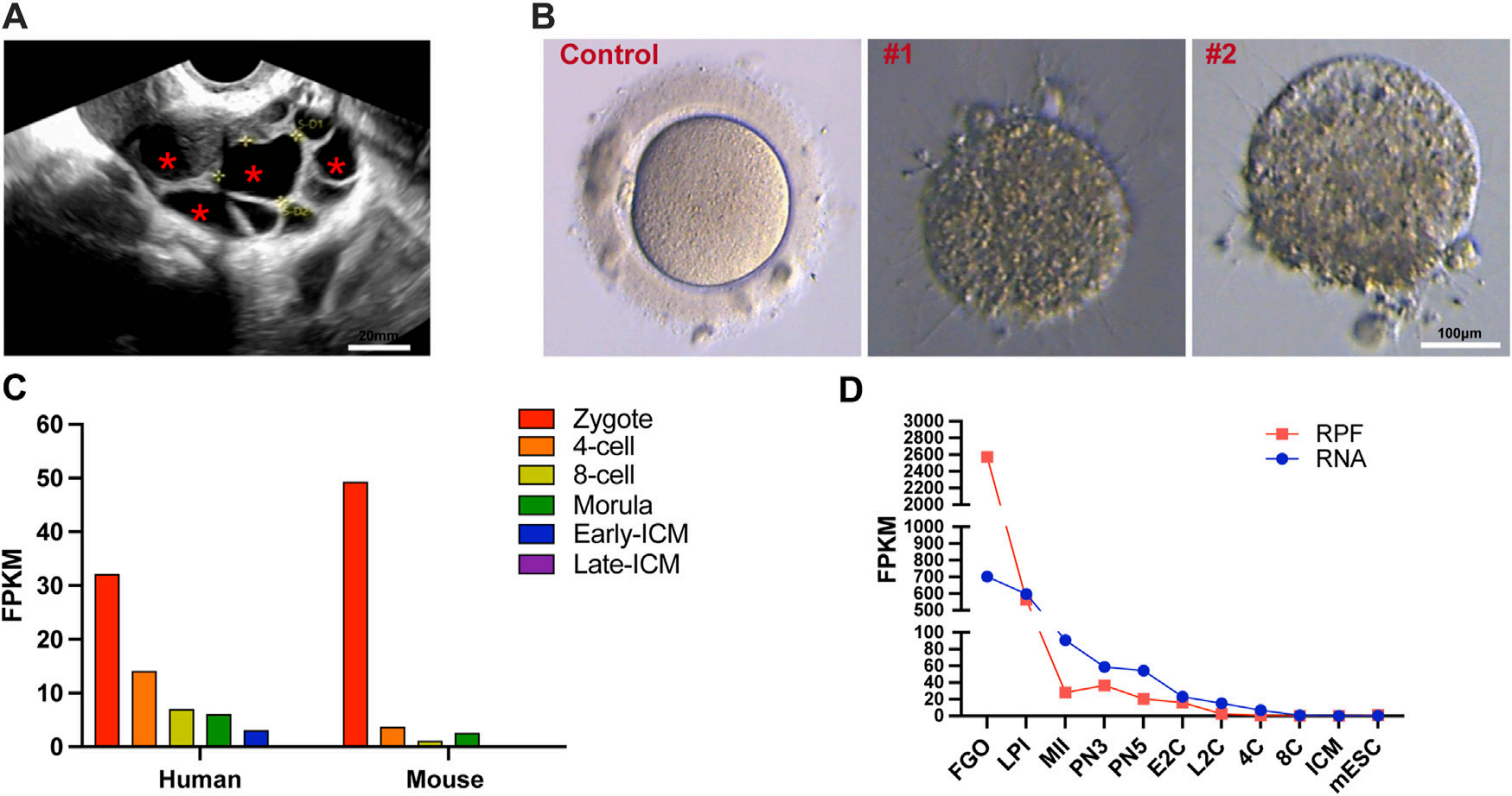
Morphological features of *ZP1* mutant follicles and dynamic expression of *ZP1* gene in different species. **(A)** Ultrasound images of *ZP1* mutant follicles as representative samples. The follicles within the cavity are indicated by "*". Scale bar is 20 μm. **(B)** The image presented depicts representative pictures of control group oocytes and patient oocytes with zona pellucida defects. Scale bar is 100 µm. **(C)** Single-cell RNA sequencing (scRNA-seq) transcriptome analysis depicting the dynamic expression of *Zp1/ZP1* mRNA from fertilization to inner cell mass (ICM) stage in mouse and human embryos. **(D)** Dynamic changes in ribosome-associated RNA expression (RPF) of *Zp1* mRNA from oocyte stage to ICM stage in mouse. The RPF line graph represents changes in RNA molecules bound to ribosomes using low-input Ribo-seq (Ribo-lite). The RNA line graph represents conventional mRNA sequencing (mRNA-seq). RPF refers to ribosome-protected fragments. Other abbreviations include FGOs (fully-grown oocytes), LPI (late prophase I), MII (metaphase II), PN3 (early one-cell stage), PN5 (late one-cell stage), E2C (early 2-cell stage), L2C (late 2-cell stage), 4C (4-cell stage), 8C (8-cell stage), ICM (inner cell mass), and mESC (mouse embryonic stem cells).

Upon presentation, a 28-year-old female patient with a 2-year history of primary infertility was admitted to our center. There were no obvious abnormalities in her reproductive organs, including the ovaries and uterus, and we can observe multiple large follicles (Figure 1A). The patient exhibited abnormal oocyte development, with over 10 follicles observed after hormonal stimulation, but only three oocytes could be retrieved. Zona pellucida-deficient oocytes, lacking the transparent layer, were observed in all specimens (Figure 1B). All her hormone levels were within the normal limits, including FSH and AMH. It has been shown by routine semen analysis that her husband has normal fertility potential. Furthermore, the absence of reproductive disorders in the patient’s family lineage over two consecutive generations, coupled with the lack of siblings, suggests that her condition appears to be isolated.

### Expression of the human *ZP1* and mouse *Zp1* gene

The expression of the human *ZP1* gene is highly pronounced in one-cell embryos with two pronuclei (2PN), and the level of *ZP1* expression gradually diminishes. Notably, in mice and human, *Zp1*/*ZP1* expression is most prominent in 2PN-embryos during various stages of pre-implantation embryo development (Figure 1C) (E-MTAB-7078) (Boroviak et al., 2018). Given the substantial storage of maternal mRNA in oocytes, the ribosome-bound mRNA provides a more comprehensive representation of protein expression alterations. To investigate the dynamic changes of *Zp1* during mouse oocyte meiosis, we reanalyzed the recently published ribosome profiling sequencing (Ribo-seq) data (GSE165782) (Xiong et al., 2022). This publicly available Ribo-seq data provides expression levels of mRNA and ribosome-bound RNA at different stages, ranging from oocytes to blastocysts in mice. The analysis reveals that the expression of *Zp1* mRNA decreases by approximately 11-fold from the oocyte (FGO) to the 2-pronuclear zygotes (PN3) (Figure 1D).

### Effect of ZP1-Y262C mutation on predicted structure

**FIGURE 2:**
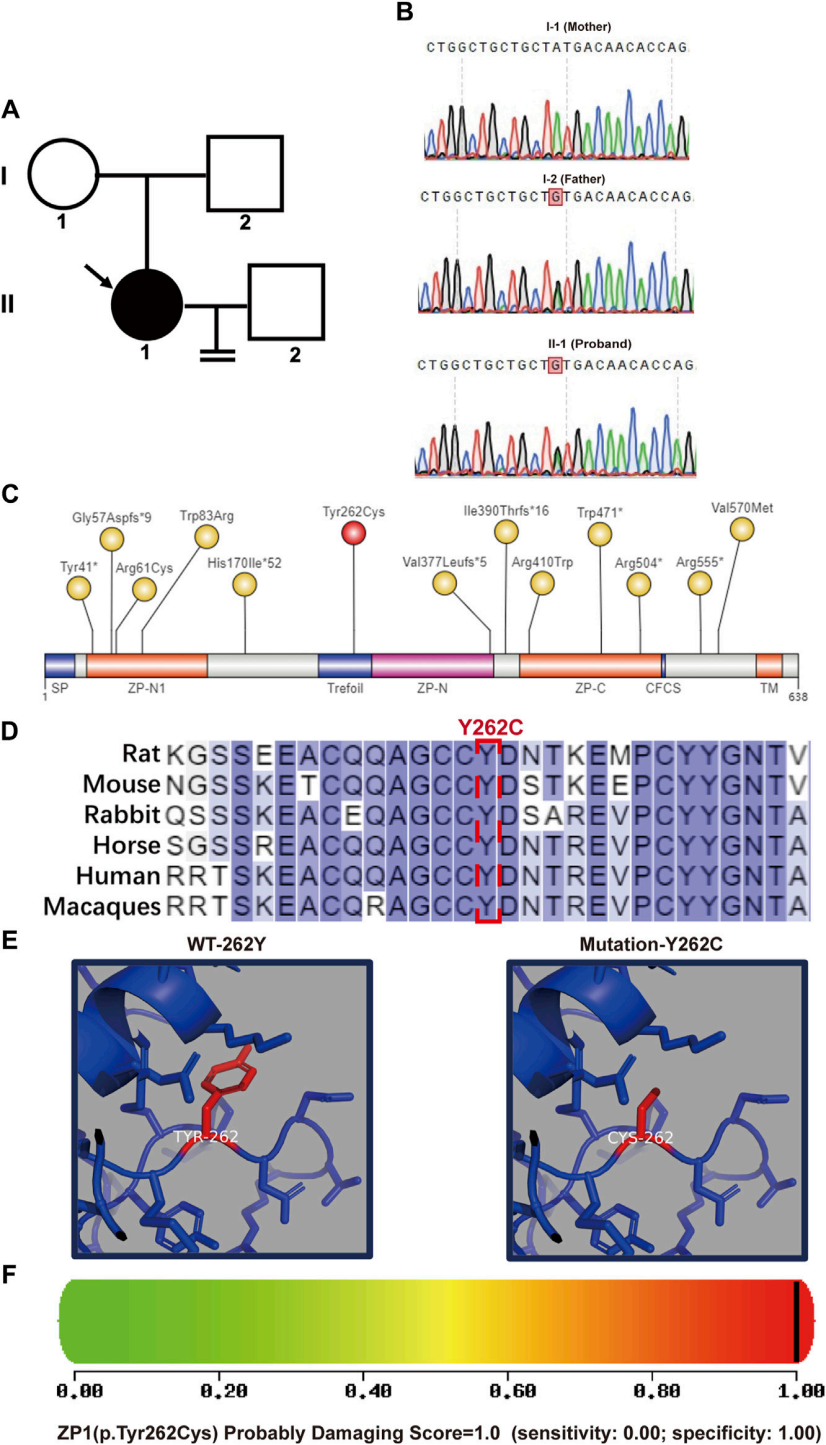
Pedigree and bioinformatics analysis of the pathogenic mutation. **(A)** Left panel shows the pedigree of the patient’s family with an arrow indicating the patient. **(B)** DNA sequencing results demonstrates that the father(I-2) and the patient (II-1) are heterozygous at nucleotide 785 (A>G) of the *ZP1* gene (NM_207341.4). Heterozygous variation of *ZP1* (NM_207341.4, **(C)**785A>G (p.Y262C)) is highlighted. **(C)** Schematic representation of the location of the mutation site within the ZP1 structural domains. Previously reported sites are highlighted in yellow, while the newly identified Y262C mutation in this study is marked in red. The ZP domain represents the zona pellucida domain. **(D)** Residue Y262 in ZP1 protein is highly conserved across six species. Human-specific sites are highlighted in red, with Y262 position indicated in a box. **(E)** Simulated spatial structure models of wild-type and mutant ZP1 protein using SWISS-MODEL software. **(F)** The potential impact of the Y262C variant on the structure and function of human ZP1 protein was predicted using PolyPhen-2. The mutation was predicted to be potentially damaging with a score of 1.000 (sensitivity: 0.00; specificity: 1.00).

In a patient (II-1) displaying abnormal zona pellucida formation, we successfully identified the heterozygous *ZP1* mutation (*ZP1*-Y262C) via whole-exome sequencing. Subsequent exome sequencing of the patient’s father (I-2) confirmed his carrier status for the *ZP1* mutation, while the mother (I-1) exhibited a normal genotype (Figures 2A,B). The *ZP1*-Y262C mutation specifically affects the trefoil domain, a compact structure composed of three intramolecular disulfide bonds formed by six cysteine residues. Infertility-associated mutations in hZP1 have been reported at a minimum of 13 distinct sites, each located within different structural domains of the protein (Figure 2C). Notably, the *ZP1*-Y262C mutation exhibits conservation across six representative species, including human, mouse, macaque, rat, rabbit and horse (Figure 2D). Differing from previously reported mutation sites, our newly identified mutation occurs at the 262nd amino acid residue, specifically situated within trefoil domain of the ZP1 protein. This novel mutation results in the substitution of tyrosine (Tyr), a phenolic group, with cysteine (Cys), which contains a thiol group (-SH). Consequently, the mutation at the 262nd residue impacts the hydrophobic interactions and polarity of the amino acid, leading to alterations in the protein structure of ZP1 (Figure 2E). Importantly, PolyPhen-2 tool predicts the severe structural damage caused by this mutation, with scores approaching 1.000 (PolyPhen-2 predicted a damaging effect with a score of 1.000 and a specificity of 1.00) (Figure 2F).

### Effects of *ZP1*-Y262C mutation on ZP1 protein expression

**FIGURE 3:**
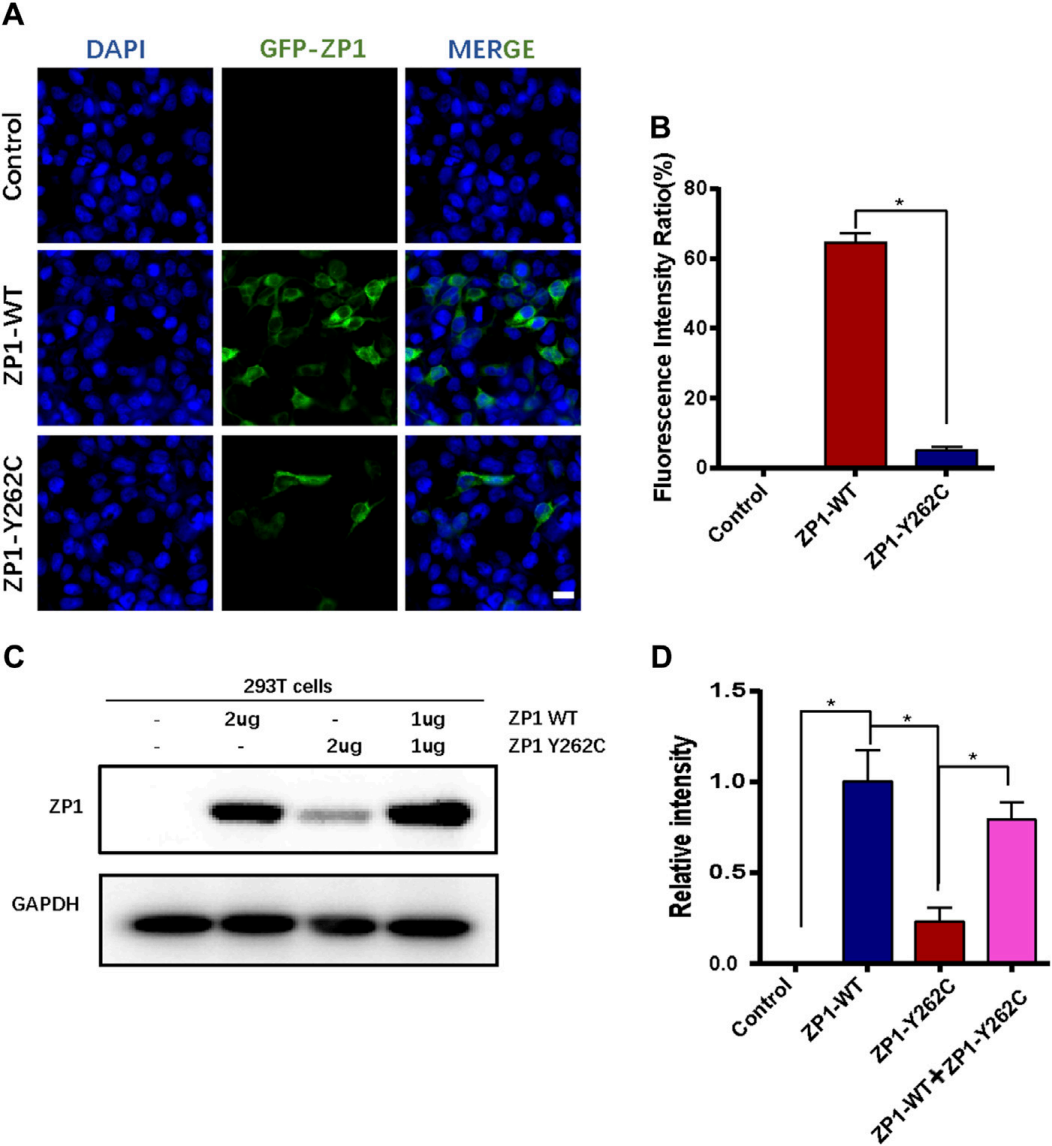
*ZP1*-Y262C mutation decreases the expression of ZP1 protein. **(A)** Localization of *ZP1* wild-type and *ZP1*-Y262C mutant proteins in the cell line. Scale bar is 20 µm. **(B)** Proportion of *ZP1* wild-type and *ZP1*-Y262C mutant positive cells. **(C)** Western blot experiment validating the expression of *ZP1* wild-type and *ZP1*-Y262C mutant proteins. Significant reduction in the expression of *ZP1*-Y262C mutant protein is observed. **(D)** Quantitative grayscale analysis of the western blot experiment in Fig3C.

The impact of the specific mutation on ZP1 protein expression was evaluated by transfecting HEK293T cells with wild-type and mutant expression plasmids. Immunofluorescence analysis indicated a significant reduction in the number of *ZP1*-Y262C mutant protein-positive cells compared to equimolar transfection with wild-type plasmids (Figures 3A,B). To further validate the effect of the *ZP1*-Y262C mutation on ZP1 protein expression, Western blot analysis was conducted (Figures 3C,D). These findings provide evidence that the *ZP1*-Y262C protein mutation leads to a reduction in ZP1 protein expression.

## Discussion

Surrounding the oocyte and early embryo, the zona pellucida (ZP) constitutes a multi-layered glycoprotein matrix, ensuring their protection. It plays a crucial role in ensuring recognition between human oocytes and sperm, preventing the entry of sperm from other species. Upon binding to the ZP, sperm can undergo reactions that facilitate penetration into the oocyte. Once a sperm enters the oocyte, the ZP functions to block the entry of other sperm (Litscher and Wassarman, 2020a). However, various issues related to the ZP are commonly observed in clinical settings, including thinning, excessive thickness, dark coloration, appearance of separations, serrations, gel-like consistency, or complete absence of the ZP. Genetic mutations in the ZP proteins (ZP1-4) can lead to ZP thinning or loss, resulting in infertility in patients (Mannikko et al., 2005; Dai et al., 2019; Yuan et al., 2019; Cao et al., 2020; Luo et al., 2020; Okutman et al., 2020; Wassarman and Litscher, 2021; Loeuillet et al., 2022; Shen et al., 2022; Pujalte et al., 2023).

In this paper, whole-exome sequencing was conducted on a patient with characteristic oocyte ZP deficiency following hormonal stimulation. Subsequent analysis led to the identification of a novel heterozygous mutation (*ZP1* NM_207341.4:c.785A>G (p.Y262C)) situated within the trefoil domain of the ZP1 protein. Further bioinformatic analysis and functional experiments confirmed that this heterozygous *ZP1* mutation led to the dysfunction of ZP1 proteins.

ZP1 plays a crucial role in cross-linking the long fibers that form the matrix of the zona pellucida (ZP). The presence of ZP1 protein is essential for maintaining the structural integrity of the ZP in mice. The N-terminal region of ZP1 in both mice and humans is rich in proline (Pro), providing flexibility to the elasticity of the ZP (Litscher and Wassarman, 2020b). Female mice with *Zp1* gene deletion exhibit a reduced number of two-cell embryos compared to the normal control group, and the number of offspring born from *Zp1*-deficient mice is significantly decreased, leading to decreased fertility (Rankin et al., 1999). Some points mutations in the ZP1 protein have been identified to cause functional impairments of ZP1 (Jovine et al., 2005; Litscher and Wassarman, 2020a; Luo et al., 2020; Wassarman and Litscher, 2021; Wu et al., 2021; Loeuillet et al., 2022) (Table 1). Mutations in the *ZP1* gene are commonly observed as homozygous or compound heterozygous mutations, with some cases showing heterozygous mutations. Mutations in the *ZP1* gene can lead to two abnormal oocyte phenotypes: zona pellucida deficiency and empty follicle syndrome (EFS). The novel mutation we present in this study has been identified in the trefoil domain of ZP1, similar to what were previously reported (Metwalley et al., 2020; Xu et al., 2020). Interestingly, the trefoil domain is unique to the hZP1 protein and is not found in hZP2 and hZP3. This trefoil domain is a compact structure composed of three intramolecular disulfide bonds formed by six cysteine residues (Thim, 1997; Tomasetto et al., 1990). The trefoil domain has also been found in two intracellular enzymes, lysosomal α-glucosidase and sucrase-isomaltase (Thim, 1997; Tomasetto et al., 1990), suggesting its potential role in carbohydrate binding. In addition, mounting evidence suggests that the trefoil domain primarily serves a structural function rather than binding to sperm (Braun et al., 2009).

**TABLE 1:** Reported mutated sites of the *ZP1* gene.

No	Sequence variation	Amino acid change	Zygosity	Phenotype	Ref.
1	c.1169_1176delTTTTCCCA	p.I390fs404X	Comp Het	No ZP	Huang et al. (2014)
2	c.123C>A	p.Tyr41Ter	Comp Het	EFS	Dai et al. (2019)
3	c.170_174delGCCAG	p.Gly57Aspfs8	Comp Het	EFS	Sun et al. (2019)
4	c.181C>T	p.Arg61Cys	Comp Het	EFS	Yuan et al. (2019)
5	c.508del	p.His170IlefsTer52	Comp Het	EFS	Dai et al. (2019)
6	c.1430 + 1G>T	p.C478Ter	Comp Het	EFS	Zhou et al. (2019)
7	c.1573-2A>G	NA	Comp Het	EFS	Dai et al. (2019)
8	c.1663C>T	p.Arg555Ter	Comp Het	EFS	Dai et al. (2019)
9	c.1775-8T>C	p.D592Gfs9	Comp Het	EFS	Zhou et al. (2019)
10	c.1127_1128del	p.Ala376GlyTer386	Comp Het	EFS	Zhang et al. (2020)
11	c.2T>A	p.M1K	Comp Het	EFS	Liu et al. (2020)
12	c.239G>A	p.Cys80Tyr	Comp Het	EFS	Luo et al. (2020)
13	c.241T>C	p.Tyr81His	Comp Het	EFS	Luo et al. (2020)
14	c.507del	p.His170Ilefs52	Comp Het	EFS	Luo et al. (2020)
15	c.199G>T	p.E67Ter	Comp Het	EFS	Wu et al. (2021)
16	c.325C>T	p.R109C	Comp Het	EFS	Wu et al. (2021)
17	c.1815_1825delGGTCCTT	p.V606Afs42	Comp Het	EFS	Wu et al. (2021)
18	c.247T>C	p.W83R	Het	EFS	Yang et al. (2017)
19	c.326G>A	p.Arg109His	Het	No ZP	Cao et al. (2020)
20	c.800_801delAG	p.E267Gfs80	Het	EFS	Wu et al. (2021)
21	c.1413G>A	p.W410X	Hom	No ZP	Yang et al. (2017)
22	c.1014 + 1G>A	NA	Hom	EFS	Dai et al. (2019)
23	c.1129_1130del	p.Val377LeufsTer5	Hom	EFS	Dai et al. (2019)
24	c.1228C>T	p.Arg410Trp	Hom	No ZP	Zhou et al. (2019)
25	c.1510C>T	p.Arg504Ter	Hom	EFS	Dai et al. (2019)
26	c.1708G>A	p.Val570Met	Hom	No ZP	Zhou et al. (2019)
27	c.769C>T	p.Q257*	Hom	EFS	Xu et al. (2020)
28	c.1112 + 1G>T	NA	Hom	EFS	Liu et al. (2020)
29	c.1775-3C>A	NA	Hom	No ZP	Okutman et al. (2020)
30	c.1097G>A	p.Arg366Gln	Hom	No ZP	Loeuillet et al. (2022)

Note: NA, represents protein alterations that have not been reported; EFS, refers to Empty Follicle Syndrome; ZP, indicates zona pellucida; * indicates a premature STOP, codon.

To investigate the impact of the *ZP1*-Y262C mutation, we conducted transfection experiments in HEK-293T cells. Our findings demonstrated a significant decrease in *ZP1* expression due to this mutation (Figure 3). Previous studies by Yang et al. have reported similar results, showing that the variants c.508delC (p.H170Ifs*52) and c.G199T (p.E67X) of *ZP1* significantly reduced the protein expression level (Yang et al., 2021). We speculate that the *ZP1*-Y262C mutation leads to the decreased expression of ZP1 through nonsense-mediated mRNA decay (NMD) or protein instability. Nonetheless, further investigations are necessary to elucidate the underlying mechanisms responsible for the reduced expression of ZP1 protein caused by this mutation.

The present study provides valuable insights into the contribution of the *ZP1*-Y262C mutation to oocyte growth. Nonetheless, certain limitations should be acknowledged. Firstly, the sample size utilized in this study is relatively small, necessitating the validation of our results by confirming the presence of similar phenotypes at the same mutation site across multiple reproductive centers. Secondly, inherent limitations in the process of exome sequencing analysis and the continuous updates in pathological variation databases need to be taken into consideration. Consequently, it is possible that our study may have overlooked the identification of other pathological variations in different genes that could be associated with this phenotype.

In conclusion, a previously unreported heterozygous mutation in the *ZP1* gene was identified, resulting in the absence of the zona pellucida in oocytes. This mutation, situated within the distinctive trefoil domain of ZP1, disrupts proper ZP assembly and oocyte maturation. These discoveries shed light on the pathogenic nature of the mutation and offer potential implications for the diagnosis and management of female infertility.

## Data Availability

The DNA sequence data presented in the study are deposited in the National Genomics Data Center (NGDC) repository, accession number PRJCA021182. Further inquiries can be directed to the corresponding author.

## References

[B1] Avella M. A. Baibakov B. Dean J. (2014). A single domain of the ZP2 zona pellucida protein mediates gamete recognition in mice and humans. J. Cell Biol. 205, 801–809. 10.1083/jcb.201404025 24934154 PMC4068139

[B2] Boroviak T. Stirparo G. G. Dietmann S. Hernando-Herraez I. Mohammed H. Reik W. (2018). Single cell transcriptome analysis of human, marmoset and mouse embryos reveals common and divergent features of preimplantation development. Development 145, dev167833. 10.1242/dev.167833 30413530 PMC6240320

[B3] Braun B. C. Ringleb J. Waurich R. Viertel D. Jewgenow K. (2009). Functional role of feline zona pellucida protein 4 trefoil domain: a sperm receptor or structural component of the domestic cat zona pellucida? Reprod. Domest. Anim. 44 (Suppl. 2), 234–238. 10.1111/j.1439-0531.2009.01370.x 19754576

[B4] Cao Q. Zhao C. Zhang X. Zhang H. Lu Q. Wang C. (2020). Heterozygous mutations in ZP1 and ZP3 cause formation disorder of ZP and female infertility in human. J. Cell Mol. Med. 24, 8557–8566. 10.1111/jcmm.15482 32573113 PMC7412702

[B5] Carino C. Diaz L. Mendez I. (2001). Zona pellucida antigens in the human ovum: its importance in contraceptive strategies. Rev. Invest. Clin. 53, 174–180. 11421113

[B6] Dai C. Chen Y. Hu L. Du J. Gong F. Dai J. (2019). ZP1 mutations are associated with empty follicle syndrome: evidence for the existence of an intact oocyte and a zona pellucida in follicles up to the early antral stage. A case report. Hum. Reprod. 34, 2201–2207. 10.1093/humrep/dez174 31734689

[B7] Eppig J. J. O’Brien M. Wigglesworth K. (1996). Mammalian oocyte growth and development *in vitro* . Mol. Reprod. Dev. 44, 260–273. 10.1002/(SICI)1098-2795(199606)44:2<260::AID-MRD17>3.0.CO;2-6 9115726

[B8] Hasegawa A. Koyama K. (2007). Contribution of zona proteins to oocyte growth. Soc. Reprod. Fertil. Suppl. 63, 229–235. 17566276

[B9] Huang H. L. Lv C. Zhao Y. C. Li W. He X. M. Li P. (2014). Mutant ZP1 in familial infertility. N. Engl. J. Med. 370, 1220–1226. 10.1056/NEJMoa1308851 24670168 PMC4076492

[B10] Jovine L. Darie C. C. Litscher E. S. Wassarman P. M. (2005). Zona pellucida domain proteins. Annu. Rev. Biochem. 74, 83–114. 10.1146/annurev.biochem.74.082803.133039 15952882

[B11] Jovine L. Qi H. Williams Z. Litscher E. Wassarman P. M. (2002). The ZP domain is a conserved module for polymerization of extracellular proteins. Nat. Cell Biol. 4, 457–461. 10.1038/ncb802 12021773

[B12] Kalia S. S. Adelman K. Bale S. J. Chung W. K. Eng C. Evans J. P. (2017). Recommendations for reporting of secondary findings in clinical exome and genome sequencing, 2016 update (ACMG SF v2.0): a policy statement of the American College of Medical Genetics and Genomics. Genet. Med. 19, 249–255. 10.1038/gim.2016.190 27854360

[B13] Litscher E. S. Wassarman P. M. (2020a). Zona pellucida genes and proteins and human fertility. Trends Dev. Biol. 13, 21–33. 33335361 PMC7743998

[B14] Litscher E. S. Wassarman P. M. (2020b). Zona pellucida proteins, fibrils, and matrix. Annu. Rev. Biochem. 89, 695–715. 10.1146/annurev-biochem-011520-105310 32569527

[B15] Liu C. Litscher E. S. Mortillo S. Sakai Y. Kinloch R. A. Stewart C. L. (1996). Targeted disruption of the mZP3 gene results in production of eggs lacking a zona pellucida and infertility in female mice. Proc. Natl. Acad. Sci. U. S. A. 93, 5431–5436. 10.1073/pnas.93.11.5431 8643592 PMC39263

[B16] Liu M. Shen Y. Zhang X. Wang X. Li D. Wang Y. (2020). Novel biallelic loss-of-function variants in ZP1 identified in an infertile female with empty follicle syndrome. J. Assist. Reprod. Genet. 37, 2151–2157. 10.1007/s10815-020-01855-x 32556881 PMC7492330

[B17] Loeuillet C. Dhellemmes M. Cazin C. Kherraf Z. E. Fourati Ben Mustapha S. Zouari R. (2022). A recurrent ZP1 variant is responsible for oocyte maturation defect with degenerated oocytes in infertile females. Clin. Genet. 102, 22–29. 10.1111/cge.14144 35460069 PMC9327729

[B18] Luo G. Zhu L. Liu Z. Yang X. Xi Q. Li Z. (2020). Novel mutations in ZP1 and ZP2 cause primary infertility due to empty follicle syndrome and abnormal zona pellucida. J. Assist. Reprod. Genet. 37, 2853–2860. 10.1007/s10815-020-01926-z 32829425 PMC7642144

[B19] Mannikko M. Tormala R. M. Tuuri T. Haltia A. Martikainen H. Ala-Kokko L. (2005). Association between sequence variations in genes encoding human zona pellucida glycoproteins and fertilization failure in IVF. Hum. Reprod. 20, 1578–1585. 10.1093/humrep/deh837 15860499

[B20] Metwalley A. Brasha N. Esteves S. C. Fawzy M. Brasha H. Hellani A. (2020). Role of diagnostic intracytoplasmic sperm injection (ICSI) in the management of genetically determined zona pellucida-free oocytes during *in vitro* fertilization: a case report. Zygote 28, 519–523. 10.1017/S0967199420000441 32847637

[B21] Okutman O. Demirel C. Tulek F. Pfister V. Buyuk U. Muller J. (2020). Homozygous splice site mutation in ZP1 causes familial oocyte maturation defect. Genes (Basel) 11, 382. 10.3390/genes11040382 32244758 PMC7231235

[B22] Picelli S. Faridani O. R. Bjorklund A. K. Winberg G. Sagasser S. Sandberg R. (2014). Full-length RNA-seq from single cells using Smart-seq2. Nat. Protoc. 9, 171–181. 10.1038/nprot.2014.006 24385147

[B23] Pujalte M. Camo M. Celton N. Attencourt C. Lefranc E. Jedraszak G. (2023). A ZP1 gene mutation in a patient with empty follicle syndrome: a case report and literature review. Eur. J. Obstet. Gynecol. Reprod. Biol. 280, 193–197. 10.1016/j.ejogrb.2022.12.011 36529558

[B24] Rankin T. Familari M. Lee E. Ginsberg A. Dwyer N. Blanchette-Mackie J. (1996). Mice homozygous for an insertional mutation in the Zp3 gene lack a zona pellucida and are infertile. Development 122, 2903–2910. 10.1242/dev.122.9.2903 8787763

[B25] Rankin T. Talbot P. Lee E. Dean J. (1999). Abnormal zonae pellucidae in mice lacking ZP1 result in early embryonic loss. Development 126, 3847–3855. 10.1242/dev.126.17.3847 10433913

[B26] Rankin T. L. O’Brien M. Lee E. Wigglesworth K. Eppig J. Dean J. (2001). Defective zonae pellucidae in Zp2-null mice disrupt folliculogenesis, fertility and development. Development 128, 1119–1126. 10.1242/dev.128.7.1119 11245577

[B27] Richards S. Aziz N. Bale S. Bick D. Das S. Gastier-Foster J. (2015). Standards and guidelines for the interpretation of sequence variants: a joint consensus recommendation of the American College of medical genetics and genomics and the association for molecular pathology. Genet. Med. 17, 405–424. 10.1038/gim.2015.30 25741868 PMC4544753

[B28] Shen Y. Guo J. Zhang X. Wang X. Zhu S. Chen D. (2022). Identification of a heterozygous variant of ZP2 as a novel cause of empty follicle syndrome in humans and mice. Hum. Reprod. 37, 859–872. 10.1093/humrep/deac026 35211729

[B29] Sun L. Fang X. Chen Z. Zhang H. Zhang Z. Zhou P. (2019). Compound heterozygous ZP1 mutations cause empty follicle syndrome in infertile sisters. Hum. Mutat. 40, 2001–2006. 10.1002/humu.23864 31292994

[B30] Thim L. (1997). Trefoil peptides: from structure to function. Cell Mol. Life Sci. 53, 888–903. 10.1007/s000180050108 9447240 PMC11147297

[B31] Wassarman P. M. Litscher E. S. (2021). Zona pellucida genes and proteins: essential players in mammalian oogenesis and fertility. Genes (Basel) 12, 1266. 10.3390/genes12081266 34440440 PMC8391237

[B32] Wu L. Li M. Yin M. Ou Y. Yan Z. Kuang Y. (2021). Novel mutations in ZP1: expanding the mutational spectrum associated with empty follicle syndrome in infertile women. Clin. Genet. 99, 583–587. 10.1111/cge.13921 33423275

[B33] Xiong Z. Xu K. Lin Z. Kong F. Wang Q. Quan Y. (2022). Ultrasensitive Ribo-seq reveals translational landscapes during mammalian oocyte-to-embryo transition and pre-implantation development. Nat. Cell Biol. 24, 968–980. 10.1038/s41556-022-00928-6 35697785

[B34] Xu Q. Zhu X. Maqsood M. Li W. Tong X. Kong S. (2020). A novel homozygous nonsense ZP1 variant causes human female infertility associated with empty follicle syndrome (EFS). Mol. Genet. Genomic Med. 8, e1269. 10.1002/mgg3.1269 32329253 PMC7336750

[B35] Yang P. Chen T. Liu Y. Hou Z. Wu K. Cao Y. (2021). The critical role of ZP genes in female infertility characterized by empty follicle syndrome and oocyte degeneration. Fertil. Steril. 115, 1259–1269. 10.1016/j.fertnstert.2020.11.003 33272616

[B36] Yang P. Luan X. Peng Y. Chen T. Su S. Zhang C. (2017). Novel zona pellucida gene variants identified in patients with oocyte anomalies. Fertil. Steril. 107, 1364–1369. 10.1016/j.fertnstert.2017.03.029 28577617

[B37] Yatsenko S. A. Rajkovic A. (2019). Genetics of human female infertility. Biol. Reprod. 101, 549–566. 10.1093/biolre/ioz084 31077289 PMC8127036

[B38] Yuan P. Li R. Li D. Zheng L. Ou S. Zhao H. (2019). Novel mutation in the ZP1 gene and clinical implications. J. Assist. Reprod. Genet. 36, 741–747. 10.1007/s10815-019-01404-1 30778819 PMC6505010

[B39] Zhang Z. Shangguan T. Li Y. He W. (2020). Loss of zona pellucida in oocytes due to compound heterozygous variants of ZP1 gene. Zhonghua Yi Xue Yi Chuan Xue Za Zhi 37, 789–791. 10.3760/cma.j.issn.1003-9406.2020.07.021 32619266

[B40] Zhou Z. Ni C. Wu L. Chen B. Xu Y. Zhang Z. (2019). Novel mutations in ZP1, ZP2, and ZP3 cause female infertility due to abnormal zona pellucida formation. Hum. Genet. 138, 327–337. 10.1007/s00439-019-01990-1 30810869

